# Biases in the Visual and Haptic Subjective Vertical Reveal the Role of Proprioceptive/Vestibular Priors in Child Development

**DOI:** 10.3389/fneur.2018.01151

**Published:** 2019-01-07

**Authors:** Luigi F. Cuturi, Monica Gori

**Affiliations:** Unit for Visually Impaired People, Science and Technology for Children and Adults, Istituto Italiano di Tecnologia, Genoa, Italy

**Keywords:** subjective vertical, vision, haptic, development, vestibular, bayesian, multisensory

## Abstract

Investigation of the perception of verticality permits to disclose the perceptual mechanisms that underlie balance control and spatial navigation. Estimation of verticality in unusual body orientation with respect to gravity (e.g., laterally tilted in the roll plane) leads to biases that change depending on the encoding sensory modality and the amount of tilt. A well-known phenomenon is the A-effect, that is a bias toward the body tilt often interpreted in a Bayesian framework to be the byproduct of a prior peaked at the most common head and body orientation, i.e., upright. In this study, we took advantage of this phenomenon to study the interaction of visual, haptic sensory information with vestibular/proprioceptive priors across development. We tested children (5–13 y.o) and adults (>22 y.o.) in an orientation discrimination task laterally tilted 90° to their left-ear side. Experimental conditions differed for the tested sensory modality: visual-only, haptic-only, both modalities. Resulting accuracy depended on the developmental stage and the encoding sensory modality, showing A-effects in vision across all ages and in the haptic modality only for the youngest children whereas bimodal judgments show lack of multisensory integration in children. A Bayesian prior model nicely predicts the behavioral data when the peak of the prior distribution shifts across age groups. Our results suggest that vision is pivotal to acquire an idiotropic vector useful for improving precision when upright. The acquisition of such a prior might be related to the development of head and trunk coordination, a process that is fundamental for gaining successful spatial navigation.

## Introduction

During development, perception of the direction of gravity (i.e., verticality) is pivotal to learn how to maintain the upright posture, the most important posture needed for locomotion and spatial navigation. In this learning process, the brain must combine information coming from different sensory modalities, crucial cues are those that signal body orientation relative to gravity (i.e., vestibular and proprioceptive) and those that inform about the orientation of objects belonging to the explored environment. Perceived verticality depends on several aspects, such as contextual information (1–3), age (4–8), and sensory loss (9, 10). In order to disclose the role of vestibular and proprioceptive sensory information on perceived verticality, much research has used a simple paradigm in which verticality is judged when tilted in the roll-plane. In this context, uni- and multisensory contributions have been investigated by focusing on the subjective visual vertical [SVV; (11–14)], the subjective haptic vertical [SHV; (15–17)] the subjective auditory vertical (18) and the interaction of visual and haptic sensory information on perceived verticality (16). The advantage of this methodology is that it provides an indirect measurement of the perceptual readout of vestibular and proprioceptive sensory information signaling body orientation relative to gravity. The upright body orientation can indeed lead to perceptual biases such as the Aubert or A-effects that indicate perceived verticality tilted toward body tilt. This effect, first discovered in 1861 (19), has been interpreted as undercompensation for body tilt driven by an idiotropic vector indicating the most common body orientation, that is upright (11). In Bayesian terms, this scenario has been expressed with a prior model that assumes unbiased vestibular and proprioceptive sensory information about body roll tilt. The percept is represented by the posterior probability distribution and can be calculated as the product between sensory information (i.e., likelihood probability distribution) and a prior peaked at the upright position (20–22). The influence of the prior has been shown to change depending on subjects' body tilt, showing that modeled sensory variability for the encoding modality of perceptual verticality increases as the tilting angle increases (21). In this context, A-effects are interpreted as the byproduct of a system that functionally improves precision around the upright orientation for small head and body tilts (22, 23). Opposite to the A-effect, the E-effect (with “E” indicating Entgegengesetzt, that is “opposite” in German) is observed when verticality estimates are biased away from body tilt (24) thus indicating overcompensation of body tilt. Such effect has been observed for tilts of a few degrees (21) and >135-150° (25, 26); a possible interpretation of the E-effect is related to how precision based on otolith sensory information varies depending on head orientation (27).

Regardless of the involved sensory modality, perception of verticality changes depending on the developmental stage. Children in scholar age (6–11 y.o.) are less precise than adults in judging visual verticality when standing upright and postural performance follows a similar pattern as it improves after 8–9 y.o. (5, 7). These findings indicate a non-negligible role of the developmental stage in gaining functional and fine balance control. However, less is known about the interaction of the balance system with other sensory modalities in this ontogenetic process.

In adulthood, the brain is able to combine sensory information provided by different sensory modalities leading to more precise estimates, for instance when combining visual with haptic (28) or with vestibular information in discrimination tasks (29–32). However, studies on children have found that multisensory integration appears later in development (33, 34), leading to different sensory weighting depending on the investigated perceptual feature. In particular, vision seems to have a prominent role in calibrating multisensory brain processes underlying object orientation discrimination (33), spatial navigation (34), and generally postural control [for a review (26)]. Relatively to the perception of verticality, the presence of vision since birth has a strong role in providing the brain with the means to build an idiotropic vector whose influence on perceived verticality is absent in congenitally blind individuals (15). With the study presented here, we intended to investigate how visual and haptic sensory readout of verticality are influenced by vestibular/proprioceptive priors across childhood. Research on priors across childhood mostly focused on within modality priors, showing developmental trends for the interaction between light from above and convexity priors (35) and more generally for lighting direction (36). To our knowledge, no studies investigated the use of priors during development on the perceptual readout of visual and haptic sensory information. To fill this gap in the literature, we took advantage of a simple object orientation discrimination task performed by subjects tilted on their left-ear side. We allowed participants to use either vision, touch or a combination of both modalities for providing the response. We tested children from 5 to 13 y.o. and adults older than 22 y.o. in order to investigate how head and body roll tilt affects visual and haptic readout of vestibular information across the main developmental stages. We found that the youngest group of children are biased in judging verticality across both modalities and in the bimodal condition showing A-effects. Older children and adults show no strong A-effects in the haptic modalities and in some cases a tendency to E-effects whereas they always show A-effects for visual judgments of verticality. Our results are nicely predicted by a Bayesian model that allows vestibular sensory information (i.e., likelihood) to vary depending on subjects' age and the prior to shifting position between upright and upside-down, thus shifting the estimate to indicate either A- or E-effects.

## Materials and Methods

### Participants

In this study, we had 90 subjects participating in the experiments. Twenty-nine children were excluded from the analysis because they could not perform the task properly as they were constantly distracted and unstill during the task (17 out of 29) or because their psychometric fit did not converge properly (12 out of 29). The remaining 61 subjects (29 females, age range 5–37 y.o.) were divided into 7 subgroups depending on the age: 6 y.o. (*n* = 6; 3 females; it includes one child of 5 y.o.); 7 y.o. (*n* = 7; 3 females); 8 y.o. (*n* = 10; 2 females); 9 y.o. (*n* = 8; 5 females); 10 y.o. (*n* = 15; 7 females); 11 y.o. (*n* = 7; 5 females; it includes 2 children of 13 y.o.); >22 y.o. (*n* = 8; 4 females; age range, 22–37 y.o.). All subjects performed the three experimental conditions except for 4 children who performed only visual and haptic conditions. All participants or their legal representatives provided signed informed consent before starting the test. This study was approved by the ethics committee of the local health service (Comitato Etico, ASL 3, Genova, Italy) and it was performed in accordance with the Declaration of Helsinki.

### Stimuli

**Figure 1:**
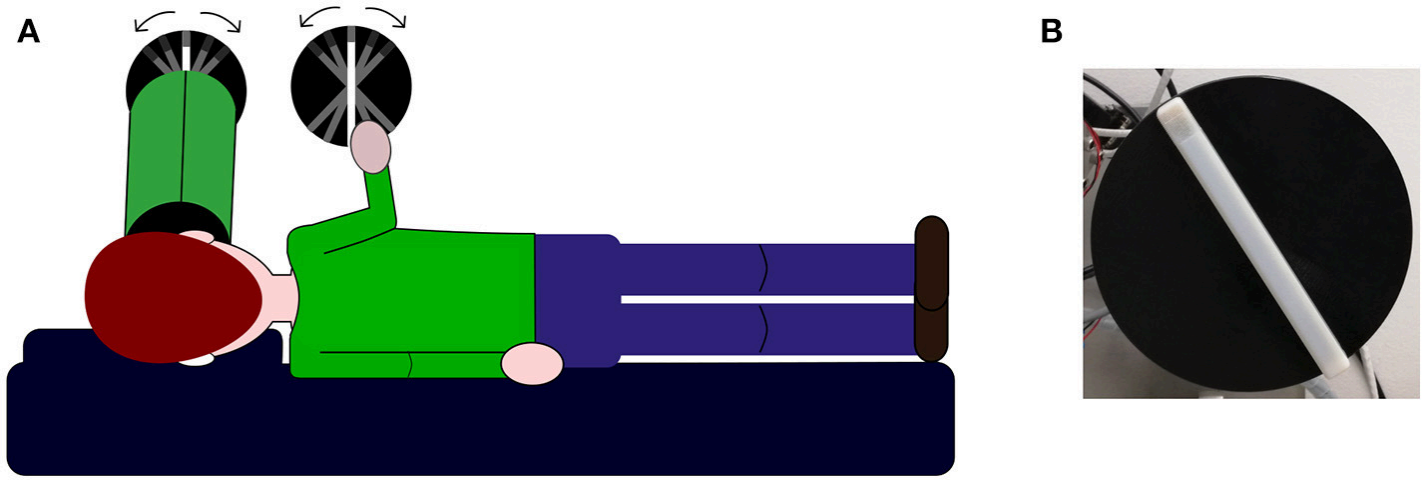
Experimental set-up. **(A)** Illustration of the set-up used to deliver visual and haptic stimuli and participants body orientation during the task. The first disk from the left represents the visual stimulus looked through a shroud represented as the green tube in front of participant's face (see methods section for details). The second disk represents the haptic stimulus and it is explored by participants with their right hand. In the bimodal condition, subjects are instructed to look through the tube and touch the haptic bar at the same time. **(B)** Picture showing the bar used in the experiments.

During the experiment, subjects laid over a memory foam matrass on their left-ear side, a pillow was added under their head in order to maintain head and body roll-tilted 90° counterclockwise relative to gravity (see Figure 1A). Two identical 3d printed white plastic bars (length: 1.5 cm; width: 1.2 cm; height: 17 cm) fixed over a black circle were used to deliver the stimuli to be judged. In both bars, a section of 2 cm at one of the bar's ends had a texture rougher than the rest of the bar thus signaling the top. Both bars were fixed over two independent computer-controlled motors. The whole experiment was controlled via MATLAB with the use of the Psychtoolbox (37). Motor's sound potentially cueing bar's rotation was masked by sound played between trials for 2.5 s; for children, we use a music theme in order to make the task amusing. The sound was played through two speakers positioned behind the setup, not visible by the participant. At the end of the played sound, the trial could start and the experimenter asked the participant to perform the verticality task to avoid any attentional and performance decay. In the visual condition, an array of LEDs was installed underneath the bar and lighten up to show the visual stimulus. Subjects viewed the luminous bar through a shroud (length, 40 cm; diameter, 12.5 cm) thus the visual stimulus subtended ~17° and no other contextual visual cue could influence the response. A blurring film was placed over the shroud's aperture close to the bar in order to blend neighboring LEDs into one luminous strip. Bar's top end was visually signaled by a dotted pattern.

### Procedure

All experiments were conducted in a darkened room. In all conditions subjects performed a two-alternative forced choice task and were asked to indicate in which direction the bar was tilted. In details, subjects were asked to tell the experimenter toward which side away from the vertical the bar was tilted by using the room's features as references (i.e., position of the window in the room was used to indicate stimulus orientation toward body tilt whereas door's position was used to indicate stimulus orientation away from body tilt). The experimenter then would record the response by pressing a key on the computer controlling bars' orientation via MATLAB. We decided to use a discrimination task because it has been shown to be less vulnerable to artifacts compared to other methods as the adjustment task (6). In all conditions, subjects were asked to look through the shroud. In the haptic condition, shroud's aperture was covered by a dark gray cardboard in order to avoid visual cues of any sort. In the haptic and bimodal conditions, the haptic bar was positioned ~40 cm away from subjects' body as this was the minimum distance allowed because of the shroud presence. In the bimodal condition, subjects were told they were touching and seeing identical bars with matching orientation and were asked to base their response on the orientation information provided by both sensory modalities. In the haptic and bimodal conditions, subjects used their right hand to explore the bar (see Figure 1). Each experimental condition was run on a single block of 100 trials for adult participants and 50 trials for children. Block order between the visual and haptic condition was alternated whereas the bimodal condition was always presented as the last block of trials in order to avoid any influence of multisensory integration processes on the unimodal conditions. Experimental blocks were presented over a period of maximum 2 days to avoid that fatigue or attentional decay could influence subjects' performance. Breaks were taken between blocks of trials.

### Psi Method

**Figure 2:**
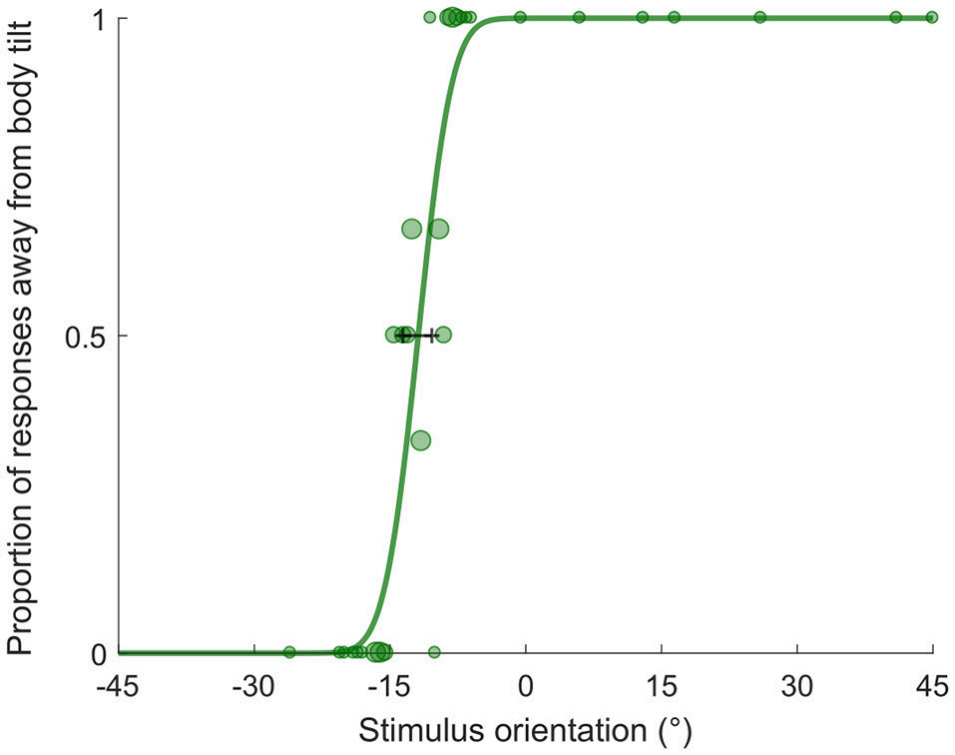
Example psychometric fit. The plot represents performance of participant aged 9 y.o. in the bimodal condition. The shift of the PSE (−11.88°) indicates the bias in perceived verticality, negative bias indicates an A-effect. The JND (2.94) represents the variability associated with the estimate. The size of dots is proportional to the number of repetitions for each stimulus value.

Stimulus orientation was determined by the PSI adaptive procedure (38), implemented using the PAL_AMPM routine from the Palamedes toolbox (39). The adaptive procedure algorithm was given an initial PSE estimate of 0° corresponding to no biased estimate. Stimulus orientation ranged between −45° and 45 degrees and changed at each trial based on the response given at the previous trial following the adaptive procedure. By using a Bayesian criterion, this method minimizes the uncertainty associated with the parameter estimates of the psychometric function (i.e., mean and standard deviation of the cumulative Gaussian fit). For each condition and subject, we fit a cumulative Gaussian to the data using the PAL_PFML_Fit routine from the Palamedes toolbox (39) which finds the best fit in a maximum likelihood sense. The point of subjective equality (PSE) is represented by mean of the distribution and it provides a measure of the orientation at which the bar is perceived to be vertical. Deviations of the PSE from 0° represents a biased estimate of verticality, therefore they will be referred as “bias” throughout the manuscript. The just noticeable difference (JND) is the standard deviation extrapolated from the cumulative Gaussian fit and it is used as a measure of precision associated with the estimate (see Figure 2).

### Statistical Analysis

In order to test whether there is an effect provided by the sensory modality used to encode stimulus orientation, either visual, haptic or bimodal, and by participants' age, we ran a linear mixed model ANOVA with the experimental condition and subjects' age (subjects are divided into 7 subgroups: up to 6, 7, 8, 9, 10, 11 y.o. and adults) as factors. The relationship between age and bias magnitude was tested by correlation analysis corrected for multiple comparisons (Bonferroni correction). *Post-hoc* analysis was conducted to test significance level in subgroups defined by age using one-tailed one sample *t*-tests corrected for multiple comparisons (Bonferroni correction). Comparison of biases between age subgroups and for each experimental condition was done by means of one-tailed paired *t*-tests corrected for multiple comparisons (Bonferroni correction). As our hypothesis predicts an A-effect for all sensory modalities in children and a reduced or absent bias in adults we used the one-tailed *t*-test that assumes biases to be >0.

### Multisensory Integration

Integration of visual and haptic sensory cues is tested by using an MLE prediction Bayesian model as previously used in several studies (28, 40). We used the following equation to calculate precision associated with the bimodal estimate:

(1) $$\begin{array}{l} \sigma_{VH}^{2}=\;\frac{\sigma_{V\;}^{2}{\;\sigma }_{H}^{2}}{\sigma_{V}^{2}+\sigma_{H}^{2}} \end{array}$$

where σ_*V*_ and σ_*H*_ are the sigma for the visual and haptic modality given by the psychometric fit respectively, and represent precision associated with the estimate. The MLE calculation assumes that the optimal bimodal estimate of the PSE (Ŝ_*VH*_) is given by the weighted sum of the independent visual and haptic estimates (Ŝ_*V*_ and Ŝ_*H*_).

(2) $$\begin{array}{l} {Ŝ}_{VH}=\;w_{V}{Ŝ}_{V}+w_{H}{Ŝ}_{H} \end{array}$$

Where each sensory modality's weight is calculated as follows:

(3) $$\begin{array}{l} w_{V}=\;\frac{{1/\sigma }_{V}^{2}}{{1/\sigma }_{V}^{2}+{1/\sigma }_{H}^{2}}\;,w_{H}=\;\frac{{1/\sigma }_{H}^{2}}{{1/\sigma }_{V}^{2}+{1/\sigma }_{H}^{2}} \end{array}$$

Two-tailed paired *t*-tests corrected for multiple comparisons (Bonferroni correction) are used in order to compare predicted accuracy and precision (i.e., PSE and JND of the psychometric fit) in children (age 6–11 y.o.) and in adults. The model predicts that when the two sensory modalities are combined, precision improves. If the model fails in predicting this pattern, e.g., by predicting higher precision than observed in the behavioral measurement, a probable explanation would be that multisensory integration is not yet accomplished.

### Bayesian Prior Model

Prediction of potential biases following the A- or the E-effects in verticality estimation were modeled by using a Bayesian modeling approach. We used this approach to test whether prior information or experience could influence the estimate of verticality depending on subject's age. Therefore, we modeled the prediction of our results based on a maximum likelihood estimation approach (MLE) where the peak of the posterior distribution represents the predicted estimate. The posterior distribution reflects the influence of the prior on the likelihood distribution which in turn represents the sensory information associated with the stimulus and it is assumed to be unbiased. We allowed three parameters to vary in order to find the best fitting prediction (in the least squares sense) to the behavioral results. The sigma of the likelihood distribution is varied depending on subjects' age by using the following Equations (4, 5). In particular, similar to previous work (21), we first calculated σ_*a*_ as:

(4) $$\begin{array}{l} \sigma_{a}\left(\rho \right)=a_{0}+a_{1}\;\rho \end{array}$$

with *a*_0_ the offset and *a*_1_ the coefficient that defines how σ_*a*_ changes with age (indicated by ρ). Based on previous behavioral results (5), we allow σ_*a*_ to vary either increasing or decreasing depending on participants' age. To this aim, *a*_1_is allowed to have positive and negative values. However, a negative sigma cannot be used in the model, therefore we adjust σ_*a*_ by using the following equation:

(5) $$\begin{array}{l} \sigma_{b=\;}\sigma_{a}+\min\left(\sigma_{a}\right)+\varepsilon \end{array}$$

with ε a constant added to avoid that the function equals 0 and the shift of the function is provided by adding the minimum of σ_*a*_. This is done to keep the decreasing relationship in case of negative values of *a*_1_ and by maintaining σ_*b*_ values always positive. Equation (5) allows to test an increasing or decreasing function whose starting point is always defined by *a*_0_and it is the same for each value of *a*_1_.

We also varied the sigma of the prior distribution (σ_*p*_) in order to test whether the prior influenced the estimate or not. Indeed, a flat prior would lead to no shifts of the posterior thus predicting no biases. Moreover, considering the controversial findings on the haptic perception of verticality showing either A- or the E-effect, we varied where the prior distribution is centered either toward or away from the upright orientation of head and body. Considering the pattern of biases as a function of age, our results seemed roughly consistent with a prior distribution that could change its peak depending on age or could maintain the same pattern of biases (A- rather than E-effects) across age. Moreover, the development of priors across age would be consistent with previous findings that show priors to depend on visual experience [(15) see Discussion]. Thus, we used four different possibilities for the prior distribution. First, as a control we used a flat prior that does not influence the estimate, thus the posterior is equal to the likelihood distribution. Second, a prior peaked at 0°, i.e., the upright position parallel to gravity that would lead to A-effects across all ages. Third, a prior peaked at 0° for children and a flat prior for adults and, fourth, a prior peaked at 0° for children and −180°, i.e., opposite to the direction of gravity thus leading to E-effects, for adults. These last possibilities assume that the prior shifts across development.

## Results

### PSE Analysis

**Figure 3:**
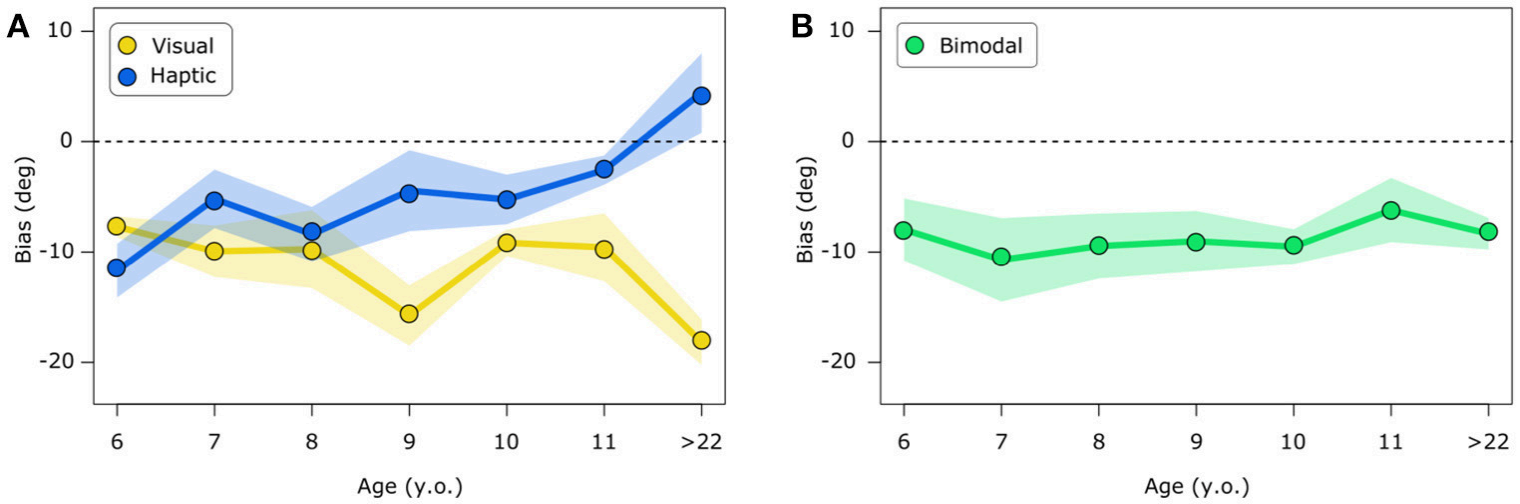
Visual and haptic biases **(A)** and bimodal biases **(B)**. Biases represent the bar's tilt in degrees at which the bar is perceived to be vertical. Positive values indicate an estimation of verticality away from body tilt, negative values represent biases toward body tilt. The first ones can be interpreted as overcompensation of body tilt whereas the latter show undercompensation of body tilt. Bimodal biases are presented separately for visualization purposes. In both figures **(A)** and **(B)**, shaded areas represent standard error.

Verticality estimates show biases depending on subjects age and the sensory modality used to encode stimulus orientation. As shown in Figure 3, in the visual condition, verticality estimates are negative, that is they are biased toward body tilt for all ages with peaks for adult participants. In the haptic condition, the youngest participants (6 y.o.) show biases toward body tilt, whereas older children show less pronounced biases and adults show a shift to the positive sign, indicating biases away from the body tilt. The bimodal condition mostly shows biases toward the body tilt both in children and adults. Linear mixed model ANOVA shows a significant effect of experimental condition [*F*_(2, 106)_ = 15.13, *p* < 0.0001], no significant effect given by age group [*F*_(6, 55)_ = 0.38, *p* = 0.88] and a significant interaction between condition and age group [*F*_(12, 106)_ = 3.66, *p* < 0.001].

*Post-hoc* analysis (one-tailed *t*-tests corrected for 21 comparisons) reveal significant biases in the visual condition for the following age subgroups 6 y.o. (*p* < 0.01), 9 y.o. (*p* < 0.01), 10 y.o. (*p* < 0.0001) and >22 y.o. (*p* < 0.001) and a tendency for the age of 7 y.o. (*p* = 0.056); significant biases in the haptic condition only for children aged 6 y.o. (*p* = 0.03) and a tendency for the age of 8 y.o. (*p* = 0.08); in the bimodal condition biases are significant for children aged 10 y.o. (*p* < 0.001) and for adults (*p* < 0.01). Correlation analysis shows that age and biases are not significantly correlated in the bimodal condition (rho = −0.04, *p* = 1); whereas there is a significant negative correlation between age and haptic biases (rho = 0.42, *p* < 0.01) and a tendency for a negative correlation in the visual condition (rho = −0.31, *p* = 0.07).

### Multisensory Integration

**Figure 4:**
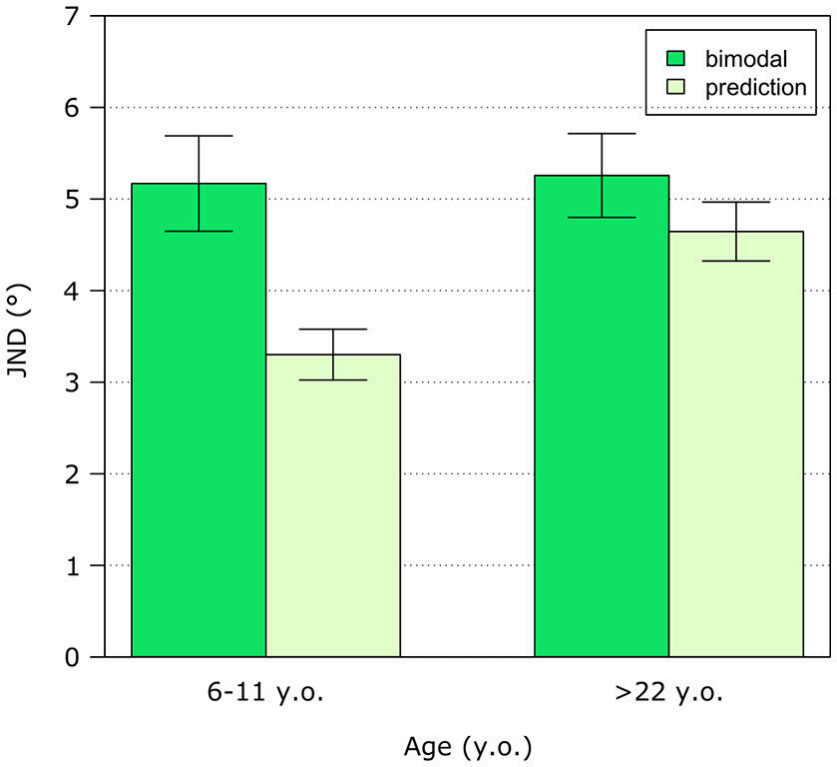
Bimodal vs. predicted variability. Variability is represented as the mean JND across subjects divided into two groups: children of primary school age (6–11 y.o.) and adults (>22 y.o.). Error bars show standard error.

Bimodal estimates of verticality show consistent biases across all ages. In the youngest group of subjects, biases are in the same direction across all conditions whereas in older children and adults biases are in between visual and haptic estimates values. The behavioral data are compared with the Bayesian integration predictions: predicted biases match the behavioral data across all ages as showed by paired *t*-tests which did not report any significant difference. Considering variability, there are no differences between adults and children for each unimodal condition that is both for the visual and haptic modality. In this case, we analyzed only the data from the subjects who did all conditions including the bimodal condition. This was done to allow us to compare bimodal and Bayesian integration prediction with the data for the unimodal conditions. The comparison between behavioral bimodal variability and the prediction shows that predicted variability matches behavioral data for the adult group (non-significant paired *t*-tests) but not for children (*p* < 0.01) (see Figure 4).

### Bayesian Prior Model

**Figure 5:**
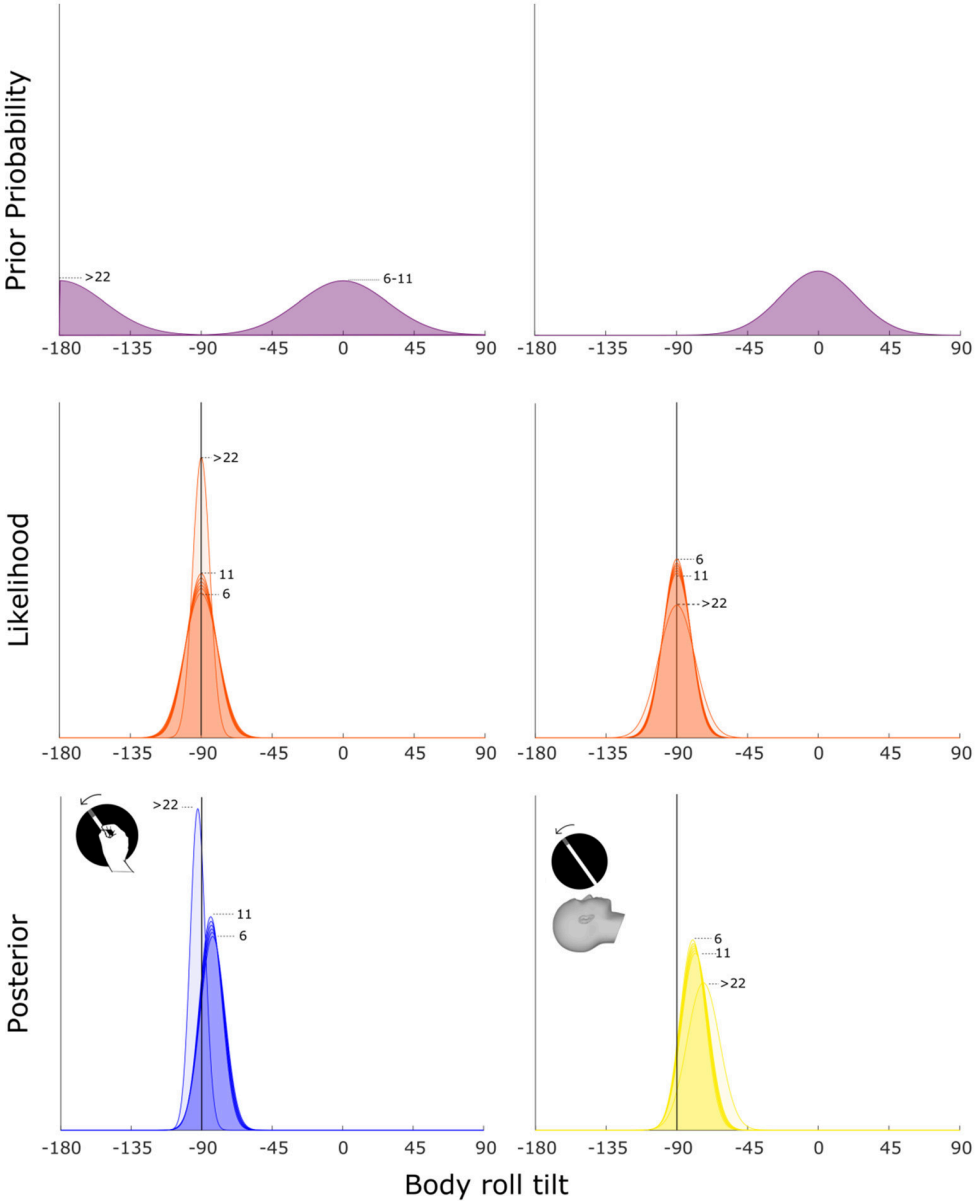
Bayesian prior model for body tilt estimation. The left and right columns show the best fitting model in a least-squares sense for the haptic and visual modality respectively. The posterior (third row) is calculated as the product between prior (first row) and likelihood (second row) distributions. The position of the posterior's peak defines whether the model predicts over- (between −180 and −90°) or undercompensation (between −90 and 0°) of body tilt. The numbers on the side of the curves indicate the age group related to each curve. For illustration purposes, age groups between 7 and 10 y.o. are not indicated.

In our model, we multiplied the likelihood with the prior in order to calculate the posterior distribution. By following the MLE approach, we consider the peak of the posterior as the predicted bias. We allowed 3 parameters to vary to find the best fitting model: where the prior is centered or a flat prior that does not influence the estimate; sigma of the prior (σ_*p*_); offset (*a*_0_) and coefficient (*a*_1_). The last two parameters (*a*_0_ and *a*_1_) are used in equation (4) to calculate the sigma of the likelihood distribution (σ_*b*_). For the visual modality, we found that the best fitting model corresponds to a prior centered at the upright position (0°) whereas in the haptic modality, the prior shifts from 0° for children and −180° for adults. Regarding σ_*p*_, variability changes depending on the encoding sensory modality, that is 24.7° for the visual modality and 28.3° for the haptic modality. We observe a different scenario regarding the parameters that define the sigma for the likelihood. For the visual modality, we observe that the best fitting offset (*a*_0_) equals 5.3 and the coefficient (*a*_1_) equals 0.16, thus indicating that a slightly increasing sigma depending on age is the one that provides the best fit (*R*^2^ = 0.1; see Figure 5). Regarding the haptic modality, we find that the best fitting offset (*a*_0_) equals 0.3 and the coefficient (*a*_1_) equals −0.16. This means that the best fit is provided by a decreasing trend of the sigma depending on participants' age (*R*^2^ = 0.18; see Figure 5).

## Discussion

In this work, we tested children and adults in a subjective vertical task. Participants were tilted on their left-hand side and had to discriminate the tilt of a bar in three conditions, visual, haptic and bimodal. Although previous research has shown effects given by gender in the perception of body orientation (41), we tested this aspect in our study and observed no influence of gender (see Supplementary Materials). Our results show biases that reflect undercompensation or overcompensation of body tilt depending on the encoding sensory modality and subjects' age. The former phenomenon is known as A-effect and in Bayesian terms can be interpreted as the influence of a prior set at the most common head and body orientation relative to gravity that is upright (i.e., an idiotropic vector). In the visual modality, bias direction is consistent at all ages showing A-effects of similar magnitude. In the haptic modality, on the other hand, the pattern of biases is modulated by subjects' developmental stage, this is confirmed by significant biases toward the tilt for the youngest group of children (i.e., 6 y.o.) as well as by a negative correlation between haptic biases and age.

In order to better understand the nature of these biases in verticality perception, we compared behavioral results with those predicted by a Bayesian model. In this context, perceptual biases have often been linked to a Bayes-optimal mechanism for which the percept depends on the influence of prior information on the readout of sensory information (42–45). Biases can indeed be interpreted as the side effect of a system that functionally takes advantages of priors in order to improve precision and generally perception. In particular, we observe that model's prediction of visual verticality is quite steady across age, that is the prior is centered at the upright position for all ages and there is a slight increase of variability in the likelihood as age increases. This happens because we did not allow prior variability to vary depending on subjects' age; therefore, we cannot exclude that prior rather than sensory variability changes with age. Regarding the haptic modality, we observed a different scenario. As we allowed the model to vary where the prior is centered with respect to participants' age, we observe that there is a shift of the prior across age, not observed instead in the visual condition. Specifically, children' biases in haptic verticality are nicely predicted by a prior centered at the upright position, thus indicating the presence of A-effects. Biases in adults, instead, are better explained by a prior centered in the opposite position, thus suggesting the presence of E-effects. However, our behavioral data do not show significant E-effects. Moreover, variability associated with sensory information about body orientation in space has a decreasing trend. These results are in line with previous findings showing that haptic orientation judgements at the early stages of development are less reliable as vision dominates for the readout of such object properties (33). In other words, we observe that haptic readout of proprioceptive and vestibular information about body orientation in space is less precise in children than in adults. In children, there is a trend to improve the precision with age and this is shown by a weaker influence of the prior as age increases.

Provided that haptic judgments of verticality have been linked to the body rather than the head reference (16, 46), our results suggest that at the early stages of development the brain is yet to disambiguate head and body references. In this sense, children are influenced by the prior as head and body are processed within the same representation of coordinates. Later in development, the two references might disambiguate thus inducing the brain to selectively access different references (e.g., priors peaked at different positions). Along these lines, previous research has shown that the ontogenesis of locomotor balance control follows a similar progression across age (47). Specifically, up to 7 years old children use an “en bloc” strategy according to which head and trunk are used as a unique block of reference frames (48). The use of such a global representation is also shown in the coordination of forearm and trunk in simple motor tasks and can be interpreted as a prominent use of egocentric reference (49). Later in development, children tend to use a different strategy by independently moving neck and trunk to maintain balance, namely an “articulated mode” (48). In this sense, the biases in perceptual verticality presented here can be considered as the byproduct of the development of a balance control system that is rougher in the youngest and it increases in complexity and articulation as age increases.

In a recent study (15), we investigated haptic perception of verticality in early and late blind adults when tilted counterclockwise. The results show that early blind individuals have no consistent biases in perceiving verticality whereas late blind subjects show an A-effect. Interestingly, such effect is not present in sighted people (see Introduction). Therefore, it is possible that the development of an idiotropic vector signaling the most important posture we need for spatial navigation might be based on the visual input at the first stages of development. Along these lines, the results reported here show that the same prior influences both visual and haptic readout of verticality at the first stages of development. As subjects' age increases, the prior maintains its influence in the visual modality whereas haptic sensory information (represented by the likelihood distribution) seems to increase in precision and the prior position might shift thus provoking a bias reduction or even inversion of bias direction, i.e., E-effects. This might represent the most important result of this research as it indicates that vision is very useful for balance control and that both haptic and visual information are used at the early stages of development to code the upright prior. However, more studies in this direction would be need to better disclose the relationship of a visually mediated prior and blindness especially if since birth. In this context, it can be proposed that a training based on haptic verticality may provide the brain with the experience necessary to build the upright prior, thus possibly improving balance and posture control in visually impaired children.

Bimodal judgments of verticality are strongly influenced by vision at all ages and Bayesian integration models do not match the behavioral data in children as previously observed [(33), for a review: (50)]. This result is true when we consider precision in verticality discrimination: children do not take advantage of the availability of both modalities to judge object orientation. Adults instead seem to be the only ones who can benefit in terms of precision in the bimodal condition. Surprisingly, although we observe no differences between adults and children when comparing precision for each individual sensory modality (i.e., vision and touch), the model predicts higher precision in the bimodal condition for children compared to adults. A slightly higher precision in one or both of the unisensory modalities for children might have led to such higher predicted bimodal precision in children. This difference might be due to individual differences in the unimodal conditions that should have been maintained also in the bimodal condition thus leading to improved precision in this condition as predicted. Since this is not the case, the reduced capability of children integrating unisensory information might underlie the observed difference between groups. On the other hand, bias prediction matches the behavioral measurement both in children and adults, thus indicating that accuracy is predicted by a Bayesian cue combination model. The reason behind the difference between precision and accuracy might rely on the fact that both visual and haptic biases in children are toward the same direction, therefore both sensory modalities are influenced by the same prior and this is maintained when both sensory modalities are available.

In adults, biases are predicted by a prior that shifts peak position depending on the involved sensory modality. This result is in line with the abovementioned lack of integration in children: on the one hand, the brain is yet to integrate the two sensory modalities, on the other hand, the sensory readout is dictated by priors that are peaked at the same body orientation, that is upright. In other words, the lack of multisensory integration and the absence of sensory specialization in possibly referring to different body coordinates (e.g., head and body) might require a similar prior to influence sensory readout to improve precision. From the model perspective, the posterior distribution representing the percept is given by the product of the prior and sensory information (i.e., the likelihood), and this product generates by definition a more skewed distribution thus representing a more precise estimate. Therefore, since precision cannot improve by multisensory integration, the brain might use a similar prior for both sensory modalities in order to maintain a functional representation of the upright, that is the most important posture the body needs to successfully move in space.

To our knowledge, our research represents the first attempt to combine Bayesian priors and multisensory integration to study the development of perception across childhood, particularly focusing on visual and haptic perceived verticality. Our findings posit visual sensory information to be pivotal not only in gaining functional perception of object orientation but also in influencing a proprioceptive/vestibular prior regarding head and body orientation relative to gravity. Moreover, we show that during the first years of development vision and touch seem to equally provide the information necessary to maintain an upright posture as both modalities are influenced by the same proprioceptive/vestibular prior. This information is useful in the context of adapting rehabilitation tools and techniques for orientation and mobility at different stages of development in people suffering of difficulties in maintaining an upright posture and avoiding falls. Rehabilitation programs may benefit from the results presented here as we show that a proprioceptive/vestibular upright prior is already acquired at the age of 6 y.o. and its influence on vision and touch depends on the developmental stage. In this sense, rehabilitation protocols might be shaped on patient's age considering the conveying sensory modality that is influenced by the upright prior, touch and vision for the youngest whereas the older ones mostly take advantage of vision.

## Author Contributions

LC and MG conceived and designed the project. LC performed experiments. LC analyzed data. LC and MG wrote and edited the manuscript. All authors gave final approval for publication.

### Conflict of Interest Statement

The authors declare that the research was conducted in the absence of any commercial or financial relationships that could be construed as a potential conflict of interest.
