# Supplementary material for: Biases in the Visual and Haptic Subjective Vertical Reveal the Role of Proprioceptive/Vestibular Priors in Child Development
Source: Front Neurol. 2019 Jan 7;9:1151. doi: 10.3389/fneur.2018.01151 (PMC6330314; doi:10.3389/fneur.2018.01151)
Supplement: Supplementary file 1 [file Data_Sheet_1.pdf]

Supplementary materials

JND analysis

Precision associated with the estimate is provided by the JND of the psychometric fit. Results are represented as average JND (in degrees) as a function of the experimental condition (see Figure S1A) and age group (see Figure S1B). Linear mixed model ANOVA shows a significant effect of experimental condition ($F(2, 106) = 24.2$, $p < 0.001$) and of age group ($F(6, 55) = 4.53$, $p < 0.001$) whereas there is no significant interaction between condition and age group ($F(12, 106) = 0.74$, $p = 0.71$). Post-hoc comparisons of precision for the effect of experimental condition are made with paired t-tests: we observe a significant difference between visual and haptic condition ($p < 0.0001$) and between haptic and bimodal condition ($p < 0.0001$) whereas precision in the visual vertical did not significantly differ from precision in the bimodal condition ($p = 1$). Paired t-tests comparing precision of each age group in all experimental conditions show that children of 6 y.o. have worse precision than subjects of 8 y.o. ($p = 0.04$), 10 y.o. ($p < 0.01$) and 11 y.o. ($p < 0.001$).

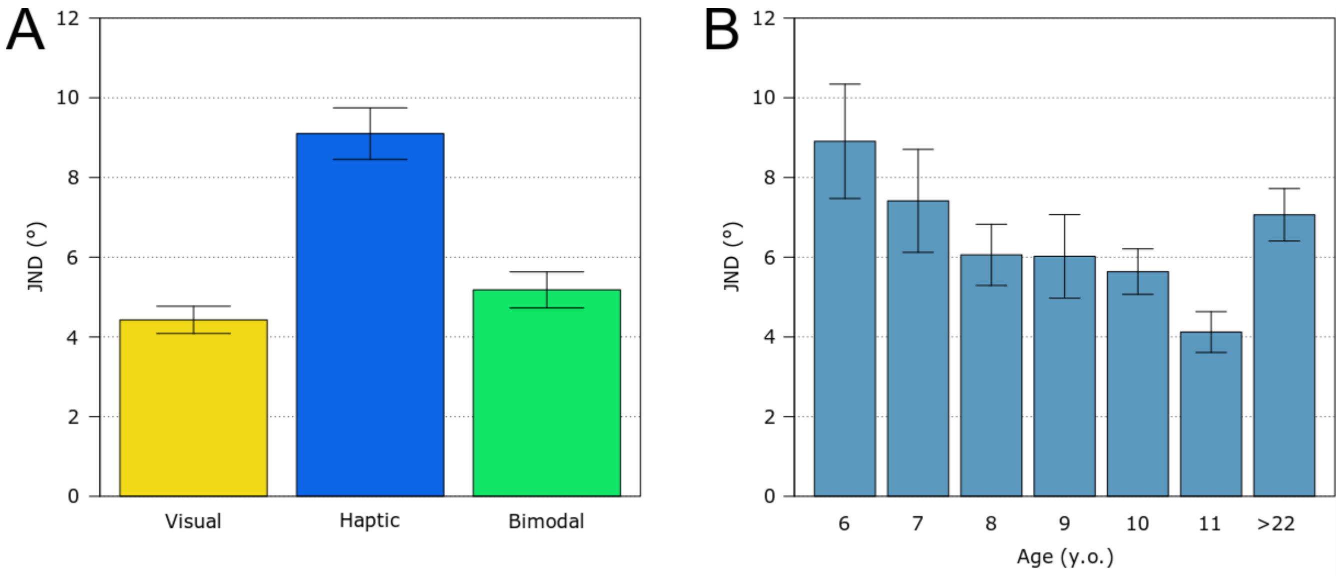

Figure S1. Precision in perceived verticality. Precision is represented as the mean JND across subjects for the three experimental conditions (A) and across age (B).

Gender effect

Considering previous observations on the effect of gender in spatial orientation perception (S 1), we additionally performed the analysis of variance by including the gender as a factor potentially influencing the bias and subjects' precision in perceiving verticality. To this end, we ran separate linear mixed model ANOVA for the bias and precision with the experimental condition, subjects' age group and gender as factors. The analysis of the bias shows a significant effect of experimental condition ($F(2, 92) = 15.1$, $p < 0.0001$), no effect by the age group ($F(6, 48) = 0.39$, $p = 0.87$) nor by gender ($F(1, 48) = 0.13$, $p = 0.71$). Moreover, the ANOVA shows a significant interaction of experimental condition and age group ($F(12, 92) = 3.69$, $p < 0.001$) and no significant interaction between the other factors (experimental condition and gender: $F(2, 92) = 1.32$, $p = 0.27$; age group and gender: $F(6, 48) = 1.77$, $p = 0.12$; experimental condition, age group and gender: $F(12, 92) = 0.87$, $p = 0.57$). The analysis of precision shows a significant effect of experimental condition ($F(2, 92) = 24.2$, $p < 0.0001$), and age group ($F(6, 48) = 4.09$, $p < 0.01$) and no effect given by gender ($F(1, 48) = 0.04$, $p = 0.83$). Moreover, the ANOVA shows no significant interaction between all factors (experimental condition and age group: $F(12, 92) = 0.73$, $p = 0.71$; experimental condition and gender: $F(2, 92) = 0.09$, $p = 0.9$; age group and gender: $F(6, 48) = 0.67$, $p = 0.67$; experimental condition, age group and gender: $F(12, 92) = 0.89$, $p = 0.55$).

Although gender is not perfectly matched for each age group, the analysis reported above suggests that the results presented in this work are not influenced by subject's gender.

Reference list

S1. Naylor YK, McBeath MK. Gender differences in spatial perception of body tilt. *Percept Psychophys* (2008) doi:10.3758/PP.70.2.199
